# Neuronal network models of epileptogenesis

**DOI:** 10.17712/nsj.2017.2.20160455

**Published:** 2017-04

**Authors:** Aminu T. Abdullahi, Lawan H. Adamu

**Affiliations:** *From the Department of Psychiatry (Abdullahi), Aminu Kano Teaching Hospital, Department of Anatomy (Adamu), Faculty of Basic Medical Sciences, Bayero University, Kano, Nigeria*

## Abstract

Epilepsy is a chronic neurological condition, following some trigger, transforming a normal brain to one that produces recurrent unprovoked seizures. In the search for the mechanisms that best explain the epileptogenic process, there is a growing body of evidence suggesting that the epilepsies are network level disorders. In this review, we briefly describe the concept of neuronal networks and highlight 2 methods used to analyse such networks. The first method, graph theory, is used to describe general characteristics of a network to facilitate comparison between normal and abnormal networks. The second, dynamic causal modelling, is useful in the analysis of the pathways of seizure spread. We concluded that the end results of the epileptogenic process are best understood as abnormalities of neuronal circuitry and not simply as molecular or cellular abnormalities. The network approach promises to generate new understanding and more targeted treatment of epilepsy.

Epilepsy is a chronic neurological condition characterised by a tendency to have recurrent, unprovoked seizures. A seizure is the transient occurrence of excessive and hypersynchronous cerebral neuronal discharge, manifesting as a clinical sign or symptom.^1^ Epilepsy is a common condition, affecting approximately 70 million people worldwide and is associated with significant morbidity and mortality.^2^ Although recurrent seizures are what define epilepsy, the condition is often associated with other morbidities in the cognitive, neurological and neuropsychiatric domains that are sometimes as important as the seizures in their impact on the quality of life of patients and the burden on their carers.^3^ These facts make epilepsy an important public health problem. The purpose of this review is to describe 2 main network models of epileptogenesis together with their respective strengths, weaknesses and clinical utilities in the management of epilepsy.

## Methods

The data bases PubMed and Science Direct were searched with the keywords “epileptogenesis” AND “neuronal network”, “epilepsy” AND “graph theory”, and “epilepsy” AND “dynamic causal modelling”. Additionally, some articles were assessed directly through Google search. Only articles written in English language were included in the review. References that were cited by the selected articles were also perused for additional material. Articles published before 2009 were excluded from the review.

### Epileptogenesis

Epileptogenesis is the process that, following some trigger, transforms a normal brain to one that produces recurrent unprovoked seizures. The trigger may be genetic or acquired and involves the development and extension of a tissue capable of generating seizures and the establishment of epilepsy or its progression, such that although its starting point may be known, the endpoint is usually obscure.^4^ The mechanisms involved are not fully understood but their study is of high importance as it may provide an opportunity for the primary prevention of epilepsy. This opportunity is most pertinent in epileptogenesis related to temporally identifiable triggers like traumatic brain injury or stroke which are common triggers that typically have significant ‘latent periods’ between the insult and the onset of habitual seizures, thus providing a window for preventive interventions.^5^ The earlier definition of seizures would suggest that the epileptogenic process will produce an imbalance between neuronal inhibition and excitation as well as neuronal hypersynchronization. An epileptogenesis model should therefore be able to demonstrate how excessive synchronization and an imbalance between excitation and inhibition occur. The other important issue with regards to epileptogenesis is whether the resulting recurrent seizures are due to molecular or cellular derangements or are the results of abnormalities in the neuronal circuitry. The neuronal network models of epileptogenesis conceive of the process as being due to the results of abnormalities in the neuronal circuitry.^6^

### Neuronal networks

A neuronal network refers to groups of neurones that form a ‘synfire’ chain in which brain structures and regions are interconnected such that activity in any one part affects activity in all other parts.^7^ The basic substrate of this interconnection is anatomical but characterization of a network must also demonstrate simultaneous co-activation or functional connectivity.^8^ Causal models are further deployed to determine the direction of influence between functionally connected elements, referred to as effective connectivity.^9^

**Figure 1:**
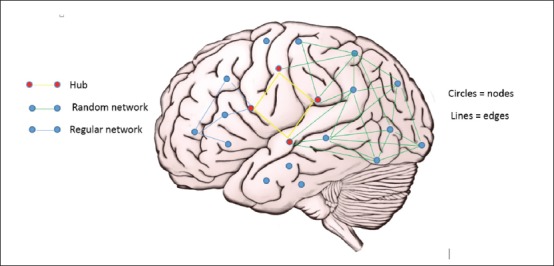
- Basic components of the neuronal network.

Computational models conceptualise the brain as a big network comprising smaller networks. Borrowing from graph theory, the models designate individual or groups of neurones are ‘nodes’ which are connected by synapses or tracts, referred to as ‘edges’. Nodes that receive extensive connections and which may be part of a number of sub networks are ‘hubs’.^10^,^11^ Network typology, which may be ‘random’, ‘regular’, ‘small world’, etc, is based on the extent and nature of connections between nodes. The normal brain exhibits small world features, which are characterized by extensive local connections but also significant long distance connections, that support local efficiency but also integration at higher levels. This model utilizes information generated from anatomical and physiological studies to characterize network changes underlying epileptogenesis and seizure transition (**Figure 1**).^12^

### Graph theory for network characterization

The recent application of network theory to neuroscience has brought new insights into understanding the relationship between brain structure and function.^13^ In the brain, structural and functional connectivity are mutually dependent, with the former shaping dynamical and informational patterns across large-scale networks, and the dynamical interactions in turn leading to alterations in the structural substrate.

In simple terms, graph theory is a mathematical framework that allows for the quantitative modeling and analysis of networks. As discussed earlier, nodes represent brain regions, while edges denote connections. The strength of an edge interconnecting 2 nodes can be derived from neuroimaging or electrophysiological studies. The systematic mapping of connectivity between all possible pairs of nodes can be used to generate a connectivity matrix, a representation that can be directly translated into graphs for network visualization and further analysis.^14^ For each node, quantitative measurements could be made that incorporate connectivity information from the complete network, reflecting the integrated nature of local brain activity. It can also result in general language that enables direct comparison of graphs that describe different types of data. Graph theory can therefore serve as a promising framework that can be used to describe how various pathological processes in neurological disorders such as epilepsy are related with each other and why the disease propagates along specific routes.^15^

**Figure 2:**
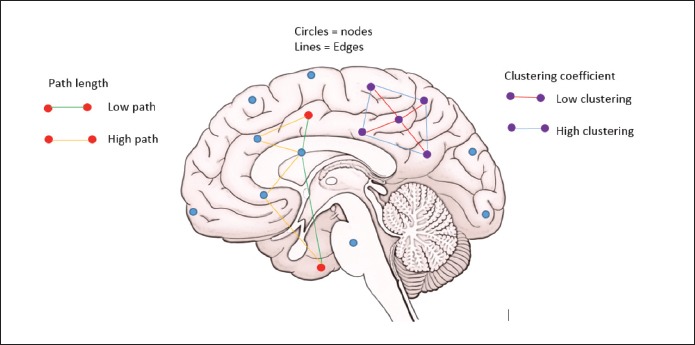
- Basic graph theoretical parameters.

Several parameters are used in neuronal network analyses using graph theory. However, the most commonly used are the clustering coefficient and the characteristic path length. Networks with a short characteristic path length can be considered globally efficient, as information needs to travel across relatively few edges from one node to another (**Figure 2**).^14^

Another important assessor of the network efficiency is ‘centrality metrics’, which can also be used to portray the relevance of each node in the network. A number of centrality parameters have been used, namely degree centrality (the overall number of connections of a given node), eigenvector centrality (a recursive formulation in which nodes that are connected to central nodes attain a high centrality score), and betweenness centrality (a measure that prioritizes nodes situated on shortest paths in the network). Centrality analysis can be used to identify hubs, which are regarded as pivotal nodes in the network. As a single centrality score can be associated with each node, the systematic mapping of centrality across brain regions can be used as an approximate mean to illustrate the connectivity organization of a given brain.^16^

In the context of epilepsy, changes in network topology were first described during the ictal period. Later, investigations focused on the interictal period and changes in network topology during this period.^17^-^21^ However, results are not similar across different studies. Some have reported an increase in clustering and a path length shortening,^17^,^19^,^20^ whereas others reported a decrease in these network properties^18^ with decreased clustering and an increased path length.^21^ These inconsistencies are probably due to different populations studied (for example, with regards to chronicity of the epilepsy), conditions (for example, under antiepileptic or drug-free conditions) and with different methodological approaches.^22^ This has left brain connectivity analysis to remain as a rapidly evolving field, with no definite consensus on optimal network construction methods and graph theoretical parameters.^14^

Despite this lack of consensus, there is an increased use of, and a promising expansion of, graph theory for clinical purposes. Graph theory has so far evolved as an important tool for quantitatively characterizing the functional architecture of the brain and has provided a new technique for assessing the structure of local and global functional connectivity in the brain.^15^,^23^ Moreover, its use in neuronal network analyses has moved research forward from studying cortical areas in isolation, by identifying why certain regions are more vulnerable based on their role in a network. If research succeeded in unifying different mapping modalities, it would be possible to have a spatiotemporally detailed view on brain connectivity in health and disease.^15^

### Dynamic causal modelling for network pathway analyses

Another method used to analyse functional connectivity in the brain is ‘dynamic causal modelling’ (DCM). This is a method which involves the interpretation of electrophysiological and functional neuroimaging data through the use of statistical parametric (coherence, correlation and covariance) mapping.^24^ It has been widely used in modelling network properties underlying specific fMRI and EEG activity.^24^-^26^ The main goal of the modelling is to study experimentally induced changes in functional integration among brain regions, which requires: (i) biophysically plausible and physiologically interpretable models of neuronal network dynamics that can predict distributed brain responses to experimental stimuli and, (ii) efficient statistical methods for parameter estimation and model comparison.^27^

There are different formulations of DCM, but they are based on a so-called “generative model”.^27^ This is a quantitative description of the mechanisms by which observed data are generated. Fundamentally, hemodynamic (fMRI) and electromagnetic (EEG/MEG) signals arise from a network of functionally segregated sources, such as brain regions or neuronal populations. This network can be thought of as a directed graph, where sources correspond to nodes and conditional dependencies among the hidden states of each node are mediated by effective connectivity, the edges.^27^ A study done based on DCM reported that slow changes in intrinsic (within-source) connectivity were required to explain seizure onset. These changes mediated a transient loss of excitatory–inhibitory balance.^28^

**Figure 3:**
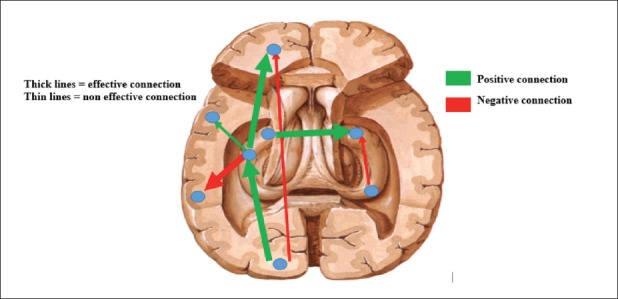
- Network pathway analyses using dynamic causal modelling.

The measured brain responses (seizure dynamics) are integrated into a generative model that incorporates a dynamic model of interacting cortical regions, and a forward model of how the neuronal activity is transformed into the measured hemodynamic response (**Figure 3**).^29^,^30^ In a study of the propagation of excitation in a genetic rat model of absence epilepsy using EEG-correlated fMRI data, DCM correctly predicted the neural driver of generalized spike-and-wave discharges, despite noticeable differences in hemodynamic delays between brain regions.^24^ This has highlighted the potential of DCM in describing certain network level disorders. In humans, DCM has been employed to investigate seizure propagation pathways based on EEG-correlated fMRI. In one such study, 2 competing hypotheses for the causal chain leading to epileptic activity propagation in a patient with a giant hypothalamic hamartomas (HH) were tested. The DCM results yielded propagation from the HH to a temporal–occipital, posterior region followed by a frontal, anterior region as the most likely model explaining the data.^31^

Model selection is strongly hypothesis-driven and the choice of the model to be tested should therefore be strictly based on the chosen hypotheses. However, since no specified model is ever exactly correct, the main driving force of model selection is to know which one, from a set of plausible alternatives, best explains the data or represents the best balance between accuracy and complexity.^32^ In a nut shell, DCM may provide important understanding into the identification of patient-specific seizure propagation pathways for pre-surgical evaluation, when a clear set of competing hypotheses exists. To support the method more direct techniques such as intracranial recordings can be used for validation purposes (**Table 1**).^33^,^34^

**Table 1:** Data sources, parameters and characteristics of the 2 models for neuronal network analyses.

Models	Data source^13^,^24^-^26^	Parameters^35^,^21^,^25^	Strenghts^17^-^21^	Weaknesses^3^,^5^	Utility^36^
Graph Theory	EEG, fMRI, Histology	i. Path length ii. Clustering coefficient iii. Centrality matrix	i. Captures ictal and interictal events as intrinsic to the network. ii. Data sets could be generated and compared with each other for validation.	i. Requires separate models for effective versus functional connectivity. ii. Analysis of effective connectivity often leads to heavy computational load	Good for assessing extent of network changes relating to the interictal state
DCM	fMRI, EEG	i. Correlation ii. Covariance iii. Coherence	Incorporates directionality and therefore, effective connectivity as a basic aspect of analysis	i. Assumes triggers of ictal and interictal events to be extraneous to the network under investigation. ii. Heavily constrained by spatial and temporal resolution of data source.	Good for identifying seizure onset zone

fMRI - Functional magnetic resonance imaging, DCM - dynamic causal modelling, EEG-Electroencephalography

### Neuronal network models and clinical epileptology

The anatomical and functional mechanisms of epilepsy may generate a piece of brain tissue with neurones of sufficiently strong and divergent connections to initiate a cascade of abnormal activity but cannot be enough to explain the clinical phenomena of seizures which necessarily involve large neuronal populations or even the whole brain. Surgically resected epileptic tissue has to be subjected to some perturbation for it to generate seizure activity. These facts would suggest that seizures are an intrinsic and dynamic property of neuronal circuits, both normal and abnormal and that they may be generated by a multitude of mechanisms.^37^

It was also suggested that the epileptogenic process utilizes the same circuits that support physiological functions to generate seizures and that what needs to be explained is how neuronal systems transit back and forth between normality and seizures. The similarity between normal thalamocortical oscillations as occur with sleep spindles and the 3 hertz spike and wave of generalized epilepsy, as well as the propensity for temporal lobe epilepsy (TLE) to take over sleep-related phenomena may be cases in point.^38^ This takeover of physiological brain circuits is so transient and occurs under very fast timescales that it is inconceivable that the brain is fundamentally different in its physico-chemical characteristics between the ictal and interictal states and would suggest that seizures are emergent properties of a complex system.^39^

### Generalised epilepsies

The generalised epilepsy is a group of epilepsy syndromes that were conceptualised as being characterised by generalized seizures. These seizures are believed to arise by the bilateral involvement of thalamo-cortical structures at onset.^1^ Recent network analyses have, however, revealed that these so-called generalized epilepsies are in fact associated with a number of focal abnormalities.^40^

Some of the most robust findings with regard to focal abnormalities in generalized epilepsy relate to abnormalities in the default mode network (DMN). Specifically, studies have documented increases in effective connectivity from the dorsolateral prefrontal cortex to the dorsal anterior cingulate cortex.^41^ The variability in the clinical presentation of the genetic generalized epilepsies like juvenile myoclonic epilepsy (JME) and childhood absence epilepsy (CAE) have also been related to whether it was cortical or thalamic structures that were involved by the disease process.^42^ In fact, there is evidence to suggest BOLD signal changes during spike-wave discharges of CAE selectively affect the precuneus and posterior cingulate gyrus, in addition to the thalamus.^43^ The selective abnormalities elucidated above have perhaps provided an explanation of the very consistent findings of neuropsychological abnormalities of frontal lobe dysfunction among patients with an ‘idiopathic’ generalised epilepsy syndrome such as juvenile myoclonic epilepsy (JME).^44^

Some of the most robust findings in idiopathic generalised epilepsy and genetic generalised epilepsy (IGE/GGE) are a general increase in connectivity, which manifested in all aspects of network analyses. This increase in connectivity was most significant in the frontal cortex, the temporal cortex and the cerebellum. Increased connectivity could serve as an explanation for the generation, or more rapid spread, of spontaneous seizures. In contrast to focal epilepsies, the gross network topology is preserved.^45^ These findings appear similar to the increased connectivity in the sensory-motor and supplementary motor cortex reported among JME patient.^46^,^47^

Modern neuroimaging techniques, such as the MRI using diffusion tensor protocols, also show the existence of focal changes in generalized epilepsies as well.^48^ The fMRI alone or in combination with routine EEG as well as specialized EEG techniques such as ‘dense array EEG’ and MEG have been deployed to explore the functional correlates of epileptogenesis in wider brain networks. Using statistical measures of correlation and coherence, they have shown that generalised epilepsies may be associated with dysfunction in similar brain networks, particularly resting state networks like the DMN, a network that underpins self-awareness.^49^,^50^ Causal models have also provided evidence of the existence of epileptogenic hubs in generalized epilepsies as well, illustrated by the precuneus in absence epilepsy.^51^ These findings have, therefore, challenged the traditional distinction between generalised and focal epilepsy and further emphasized the primary role of neuronal networks in epileptogenesis.^52^,^53^

### Focal epilepsy

Temporal lobe epilepsy (TLE) is the most common form of human epilepsy and constitutes a relatively homogeneous category within the focal epilepsy syndromes.^54^ Most studies in focal epilepsies have, therefore, paid more attention to TLE. Data on cortico-cortical networks derived from fMRI covariance show an increased clustering and path length in patients with drug-resistant TLE relative to controls. It is characterized by a topological shift suggestive of a more regular network organization, with increased covariance between sub-regions from the same structure (for example, hippocampal-to-hippocampal), and sub-regions from different structures (for example, hippocampus and amygdala).^14^ Redistribution of hub nodes in TLE have also been reported.^18^,^20^ Additionally, a high fragility of hub regions has been suggested in epilepsy.^55^

In a longitudinal fMRI covariance network analysis in 27 TLE patients, increases in path length from baseline to follow-up have been observed. This may explain the possible progressive network remodeling when the disease duration is longer. It has also been reported that pre-surgical network metrics may relate to seizure freedom after surgery.^20^,^56^ Furthermore, graph theoretical network parameters may have potential in the prediction of neurocognitive outcome after surgery.^57^ In understanding focal epilepsies, electrophysiological studies have consistently emphasized the importance of an epileptogenic network, rather than a single region,^58^,^59^ the epileptogenic network has been reported to often extend to entorhinal, lateral temporal, and inferior frontal cortices as well, in addition to subcortical nuclei, such as the amygdala and medial thalamus.^58^

The issue of altered functional connectivity beyond the temporal lobes region has been the subject of a number of studies. Connectivity maps seeding in these areas of the epileptogenic network have shown a number of abnormalities, comprising decreased connectivity within a set of sub regions in the epileptic temporal lobe,^60^-^62^ decreased connectivity between hippocampi,^62^-^65^ and decreased connectivity between the hippocampus and the orbito-frontal region.^63^

Many of these have reported abnormal interactions between mesiotemporal seeds and targets in posterior cingulate, precuneus, inferior parietal, and medial prefrontal cortices.^65^-^68^ Disruptions of connectivity in these regions involved the so-called default mode network (DMN).^69^ They may also relate to reorganization of memory circuits in this condition.^70^,^71^ Beyond mesiotemporal and DMN networks, resting-state functional connectivity disruptions in TLE have also been documented in regions known to be involved in sensory processing^72^,^73^ and attention,^73^ together with subcortical and cerebellar areas.^65^,^66^,^68^,^74^ Altogether, converging evidence from histology, electrophysiology, and neuroimaging would suggest that the pathological substrate of TLE is not restricted to the mesiotemporal lobe structures, but relates to abnormal brain structure, function, and connectivity at a systemic scale (**Table 2**).^13^

**Table 2:** Comparison between focal and general epilepsy with respect to network changes.

Syndrome type	Structures affected^1^,^41^,^43^,^65^-^68^	Network changes^14^,^62^-^65^	Implication^40^,^51^-^53^
Focal epilepsy (e.g TLE)	Lateral temporal, entorhinal, inferior frontal cortices, subcortical nuclei (the amygdala and medial thalamus), posterior cingulate, precuneus, inferior parietal, and medial prefrontal cortices	A shift from small to world configuration towards a random configuration. a shift towards a more regular network organization. Decreased connectivity between hippocampi, decreased connectivity between the hippocampus and the orbito-frontal cortex.	TLE is not restricted to the mesiotemporal lobe structures
Generalized epilepsy (e.g Childhood Absence Epilepsy)	Thalamo-cortical, Precuneus, posterior cingulate gyrus prefrontal cortex, temporal cortex and cerebellum	Focal cortical changes in precuneus precede changes in the thalamus. Increase in connectivity in the frontal cortex, temporal cortex and the cerebellum. Preservation of normal network topology	Generalized epilepsies are associated with a number of focal abnormalities.

TLE- Temporal lobe epilepsy			

### Utility, limitations and future directions of the networks models

The network models of epileptogenesis are already generating proposals for a change in the way epilepsy is classified which follows naturally from the blurring of the distinction between ‘focal’ and ‘generalised’ that the models have engendered.^53^ The models also generate a lot of expectation for a better understanding of epilepsy mechanisms and the development of more targeted treatments of epilepsy including surgery, electrical stimulation and drugs through the identification of nodes, connections and specific neurotransmitters that may be relevant to specific networks.^51^ They also raise the possibility that network plasticity mechanisms that underpin learning, for example, might also be relevant in epileptogenesis and therefore unlearning them might be feasible.^75^

Some of the controversial and perplexing phenomena associated with epilepsy, such as generalized EEG desynchronization before the onset of focal seizures, reflex seizures, as well as the precipitation of absence attacks by decreased alertness might also be more easily understood under the network models.^76^

The network models are limited by the fact that they necessarily rely on computer modelling of epleptogenesis with information gathered from neuroanatomical and functional studies, information that is often inadequate for the level of intricacy involved.^34^

The future of the network models of epileptogenesis would therefore be dependent on the development of more robust computer models as well as the mobilization of more data sources that could enhance the clinical utility of the model.

In conclusion, neuronal networks models of epileptogenesis attempt to generate a parsimonious explanation for the varied and disparate phenomena associated with epilepsy and seizures and to understand epilepsy beyond merely recurrent seizures but as dynamic property of physiologic neuronal systems. They offer the hope for better and more refined treatments for epilepsy.
